# Restoration of miR-1228* Expression Suppresses Epithelial-Mesenchymal Transition in Gastric Cancer

**DOI:** 10.1371/journal.pone.0058637

**Published:** 2013-03-12

**Authors:** Litao Jia, Jia Wu, Lu Zhang, Jiamin Chen, Dandan Zhong, Song Xu, Chuangao Xie, Jianting Cai

**Affiliations:** 1 Department of Gastroenterology, Second Affiliated Hospital of Zhejiang University College of Medicine, Hangzhou, China; 2 Department of Gastroenterology, First Affiliated Hospital of Zhejiang Chinese Medical University, Hangzhou, China; 3 Department of Gastroenterology, Tongde Hospital of Zhejiang Province, Hangzhou, China; Sapporo Medical University, Japan

## Abstract

Dysregulated miRNAs play critical roles during carcinogenesis and cancer progression. In the present study, the function of miR-1228* in regulating cancer progression was investigated in gastric cancer. Decreased expression of miR-1228* was observed in human gastric cancer tissues comparing to normal tissues. Subsequently, the role of miR-1228* was evaluated *in vivo* using the tumor xenograft model. In this model, miR-1228* overexpression suppressed xenograft tumor formation. Furthermore, we demonstrated miR-1228* negatively regulated NF-κB activity in SGC-7901 gastric cancer cells and found that CK2A2 was a target of miR-1228*. Upregulation of miR-1228* decreased the expression of mesenchymal markers and increased the epithelial marker E-cadherin, suggesting its potential role in suppressing epithelial-mesenchymal transition. Collectively, these findings provide the first evidence that miR-1228* plays an important role in regulating gastric cancer growth and suggest that selective restoration of miR-1228* might be beneficial for gastric cancer therapy.

## Introduction

MicroRNAs (miRNAs) are a class of short (20–23 nucleotides in length), endogenous, single-stranded RNAs which regulate gene expression by causing translational repression or mRNA degradation [1], [2]. Via these molecular mechanisms, miRNAs possess normal biological functions, such as regulation of cell proliferation, differentiation, and apoptosis. Moreover, dysregulated miRNAs have been shown to play critical roles in regulating carcinogenesis and cancer progression [3], [4]. Abberant miRNAs expression have been observed in many kinds of malignancies including gastric cancer [5].

Epithelial-mesenchymal transition (EMT) is a biologic reprogramming event that allows epithelial cell to undergo multiple biochemical changes to acquire a mesenchymal cell phenotype. While EMT is critical for appropriate embryonic development, increasing evidence suggests that the aberrant activation of EMT leads to malignant tumor progression [6]. It has been shown that EMT is a key process contributing to the development of cancer, characterized by the loss of the epithelial marker E-cadherin, an increase in the mesenchymal marker Vimentin, and an increase in the migratory and invasive behavior [7], [8].

In our previous study, by analyzing the miRNA array of pancreatic cancer spheres, miR-1228* was found as one of cancer-related miRNAs (data not shown). Here, we provided evidence that miR-1228* was down-regulated in gastric cancer tissues compared with normal tissues. Restoration expression of miR-1228* in gastric cancer cells significantly inhibit cell migration and tumor growth. Mechanistically, we demonstrated that miR-1228* inhibits NF-κB activation and potentially suppresses EMT.

## Materials and Methods

### Cell Culture

Human gastric cancer cell lines SGC-7901, AGS and BGC-823 were purchased from the Institute of Biochemistry and Cell Biology (Chinese Academy of Sciences). One normal gastric epithelial cell line GES-1 was purchased from the Beijing Institute for Cancer Research. These cells were maintained at 37°C in a 5% CO2 incubator in RPMI-1640 (SGC-7901 and BGC-823), F-12K (AGS) or DMEM (GES-1) medium supplemented with 10% fetal bovine serum [9], [10].

### Clinical Samples

Fifty pairs of gastric cancer and adjacent non-cancer tissue samples were obtained from patients who underwent surgical resection at the Second Affiliated Hospital, College of Medicine, Zhejiang University. The matched non-cancer adjacent tissues were obtained at least 5 cm away from the tumor site. None of the patients had undergone radiotherapy or chemotherapy prior to the surgery. Tissue samples were collected, snap-frozen in liquid nitrogen, and stored at −80°C until used [11]. All tissues were histologically confirmed to be gastric adenocarcinomas. The study was approved by the Second Affiliated Hospital Ethics Committee of Zhejiang University College of Medicine. The signed informed consent was obtained from all participants or from patients’ representatives if direct consent could not be obtained.

### RNA Extraction and Real-time PCR

Total RNA was extracted from the cultured cells or tissues using TRIzol (Invitrogen), and the concentration of total RNA was determined. Synthesis of cDNA and real-time PCR were performed using the TaqMan MicroRNA Reverse Transcription Kit (Applied Biosystems) and the TaqMan MicroRNA Assay Kit (Applied Biosystems), respectively. Real-time PCR was performed on an Applied Biosystems StepOnePlus™ Real-Time PCR System according to the protocol. The expression level of miR-1228* was normalized to RNU6B. The real-time PCR reactions were performed in triplicate. The relative expression of miRNAs was calculated using the comparative Ct method [12].

### Tumor Inoculation Assay in Nude Mice

Female BALB/c athymic nude mice at the age of 4 weeks were purchased from the Shanghai Laboratory Animal Center (Chinese Academy of Sciences). All the procedures involving animals were approved by Experimental Animal Ethics Committee, College of Medicine, Zhejiang University. miR-1228* or miR-NC stable transfection SGC-7901 cells suspensions (2.5×10^7^ cells/ml) in 200 µl serum-free medium were subcutaneously injected into the flanks of nude mice, respectively. Tumor growth was examined twice per week for 5 weeks and the tumor volume (V) was monitored by measuring the length (L) and width (W) of the tumor with calipers and calculated with the formula V =  1/2 (L×W×W) [13].

### Cell Migration

The migration ability of miR-1228* or miR-NC stable transfection SGC-7901 cells were detected using Transwells (8-mm pore size, Corning). The Transwells were put into the 24-well plates. Freshly trypsinized and washed cells were suspended in 100 µl serum-free RPMI1640 containing 1% fetal bovine serum. About 1×10^5^ cells/well was placed in the top chamber of each insert. 200 µl of RPMI1640 containing 20% fetal bovine serum was added into the lower chambers. After incubating for 24–48 h at 37°C in a 5% CO_2_ humidified incubator, cells were fixed with 95% absolute alcohol and stained with crystal violet [14]. The cells in the inner chamber were removed with a cotton swab and the cells attached to the bottom side of the membrane were counted and imaged under an inverted microscope at ×200 magnification over five random fields in each well. Each experiment was performed in triplicate.

### Western Blot

Total proteins were measured using the BCA kit (Pierce) according to the manufacturer's protocol. The proteins of cells were fractioned by electrophoresis on 12% SDS polyacrylamide gel and electro-blotted to nitrocellulose (NC) membranes for 2.5 hours at 200 V. The NC membranes were incubated with blocking buffer for 1 hour at room temperature, followed by overnight treatment with the primary antibody at 4°C, and then with an HRP-conjugated secondary antibody (MBL). After washing, the reactive bands were detected using an ECL Western blot detection kit (Millipore) according to the manufacturer’s instructions. Antibodies used in this experiment were rabbit monoclonal anti-E-cadherin (Epitomics), anti-Vimentin (Epitomics), anti-β-catenin (Epitomics), anti-Snail (Cell Signaling), anti-Slug (Cell Signaling), anti-ZEB1 (Cell Signaling), anti-ZEB2 (Santa Cruz), anti-CK2A2 (Santa Cruz) and mouse anti-GAPDH (KangChen Biotech).

### Immunohistochemistry

Paraffin sections, 3-µm in thickness, were baked for 2 h at 60°C and deparaffinized. Antigen retrieval was performed using citrate sodium buffer (PH 7.2) at 95°C for 15 minutes and then slides were cooled at room temperature for 30 minutes. After being treated with 3% hydrogen peroxide for 15 minutes to block the endogenous peroxidase, the sections were treated with normal goat serum confining liquid for 30 minutes to reduce non-specific binding and then rabbit polyclonal anti-E-cadherin (Santa Cruz) or rabbit monoclonal anti-Vimentin (Epitomics) was incubated the sections for 12 h at 4°C. After rewarming for 1 h and washing for 5 times, sections were incubated with secondary antibody for 30 minutes at room temperature. Diaminobenzidine was used for color reactions.

### Plasmid Construction and Cell Transfection

The miR-1228* expression vector (miR-1228*) or negative control (miR-NC) was constructed by cloning of annealed oligonucleotides that contained the optimized miR-1228* stem-loop (Oligonucleotides were designed as: top strand, 5′-TGC TGG TGG GCG GGG GCA GGT GTG TGG TTT TGG CCA CTG ACT GAC CAC ACA CCC CCC CGC CCA C-3′; bottom strand, 5′- CCT GGT GGG CGG GGG GGT GTG TGG TCA GTC AGT GGC CAA AAC CAC ACA CCT GCC CCC GCC CAC C-3′.) or negative control stem-loop into pcDNA6.2-GW/EmGFP vector (Invitrogen) [15]. The 2-kb miR-1228* promoter region was amplified (Primers were designed as: forward, 5′-ATC TAG GGT ACC CTC ACT TGG AG CCA CAC AGA-3′; reverse, 5′-GAC TGA AGA TCT ACC TCA AGA GTT GGG GTG TG-3′.) and coloned to pGL3-Enhancer Vector (Promega) [16], named as pGL3-miR-1228*-promoter. The empty pGL3-Enhancer Vector acted as a negative control (pGL3-control). The miR-1228* target region of the CK2A2 3’UTR sequence was chemically synthesized by Shanghai Biotech and inserted downstream of the pMIR-REPORT luciferase plasmid (Applied Biosystems), named as pMIR-CK2A2. The pReceiver-c-Rel ORF clone with GFP-tag was purchased from GeneCopoeia. To generate stable transfected cells, the miR-1228* and miR-NC expression constructs were transfected into the SGC-7901 cell line using Lipofectamine 2000 (Invitrogen) according to the manufacturer’s instructions. After 48 hours, blasticidin (14 µg/ml) was added to medium. The pGL3-miR-1228*-promoter/control, GFP-c-Rel/control, pMIR-CK2A2/control or NF-κB reporter vector (Promega) were transiently transfected into SGC-7901 cells using Lipofectamine 2000, pRL-TK (Promega) was used as internal normalization [17].

### Luciferase Reporter Assay

Luciferase reporter assay was performed in SGC-7901 cells. The cells were washed with PBS twice and lyzed in Passive Lysis Buffer (Promega) and the luciferase activities were measured from 20 µl lysate using the Dual-Luciferase Reporter Assay System (Promega) on GloMax 20/20 Luminometer (Promega). All the data were obtained by averaging the results from at least three independent repeats.

### Statistical Analysis

Differences between the expression levels of the miR-1228* in gastric cancer patients were determined by the Wilcoxon signed-rank test. The clinical data were analyzed using the chi-square test. Student’s t-test and ANOVA were employed to analyze the *in vitro* and *in vivo* data. All *P* values were two-sided and differences were defined as statistically significant for *P* < 0.05. Results were analyzed by using the SPSS V.16.0 software (SPSS Inc). * P < 0.05, ** P < 0.01.

## Results

### miR-1228* is Frequently Down-regulated in Human Gastric Cancer

**Figure 1:**
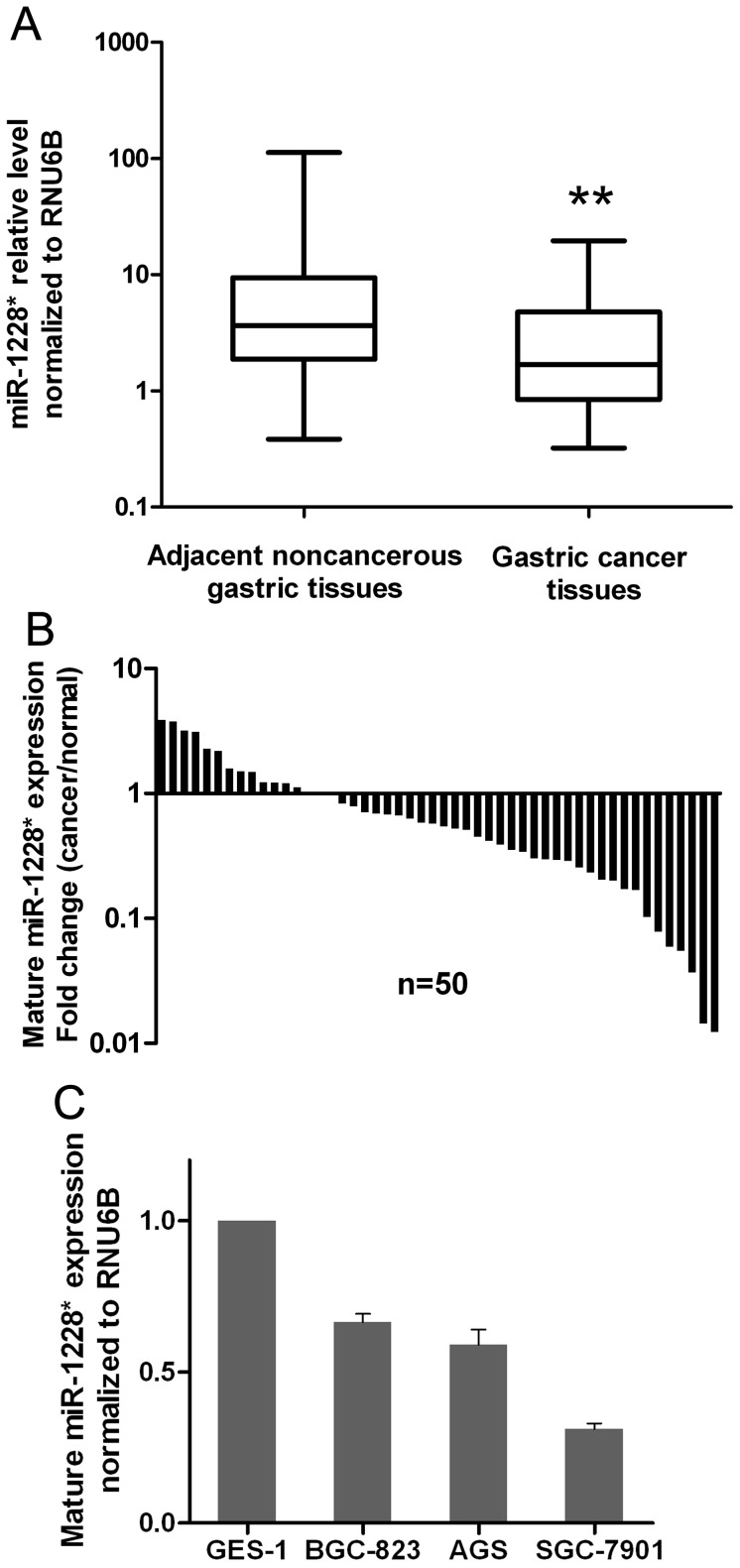
Downregulation of miR-1228* in gastric cancer tissues and gastric cancer cell lines. (A) In human gastric cancer tissues compared with paired adjacent noncancerous (normal) gastric tissues, the miR-1228* was down-regulated. The expression of miR-1228* was analyzed by real-time PCR and normalized to RNU6B. The results are displayed on a log scale. The statistical differences between samples were analyzed with the wilcoxon signed-rank test (n = 50). (B) Relative expression of miR-1228* in 50 gastric cancer tissues compared with matched normal tissues. Data are shown as 2^-ΔΔCT^ values. (C) The expression of miR-1228* in 3 gastric cancer cell lines and one immortalized normal gastric mucosal epithelial cell line (GES-1) was carried out by real-time PCR and normalized to RNU6B. Results are means ± SEM, n  =  3.

Firstly, to determine whether the miR-1228* is differentially expressed in human primary gastric cancers, the expression level of the mature miR-1228* was examined using TaqMan real-time PCR in 50 pairs of human gastric cancer tissues and pair-matched adjacent noncancerous gastric tissues. Our results showed that the expression level of miR-1228* was significantly decreased in gastric cancer tissues in comparison with the adjacent noncancerous gastric tissues (Fig. 1A). About 72% of tumor samples were lower expressed with miR-1228* (Fig. 1B). Using 2^-ΔΔCT^ values, fold change of miR-1228* < 1.0 was considered as low, while it > 1.0 was regarded as high expression [18]. miR-1228* expression was also evaluated in gastric cancer cell lines and one immortalized normal gastric mucosal epithelial cell line (GES-1). As shown in Fig. 1C, miR-1228* was significantly low expression in all cancer cell lines compared with GES-1. Taken together, these results provide strong evidence that miR-1228* was down-regulated in gastric cancer.

Furthermore, miR-1228* expression were evaluated with regards to the clinicopathological characteristics of the patients. All patients had no distant metastasis and all tissues were histologically confirmed with gastric adenocarcinomas. All cases (n = 50) were stratified into two groups: miR-1228* low expression (n = 36) and miR-1228* high expression (n = 14). The miR-1228* low-expression group had inclinations towards larger tumor size. However, there were no significant relationships between the miR-1228* expression and other clinicopathologic features such as age, gender, histological type or TNM stage (Table 1).

**Table 1:** Relationship between miR-1228* expression and clinicopathologic features of patients with gastric cancer (n = 50).

		miR-1228* expression		
Variables	Number	High (n = 14)	Low (n = 36)	*P* value
Age (years)				0.243
<60	22	8	14	
≥60	28	6	22	
Gender				0.875
Male	40	11	29	
Female	10	3	7	
Tumor size (cm)				0.018*
<5.0	33	13	20	
≥5.0	17	1	16	
Histological grade				0.240
Poor	15	5	10	
Moderate-poor	19	7	12	
Moderate	16	2	14	
Tumor invasion (T)				0.065
T1	6	4	2	
T2	6	1	5	
T3	21	3	18	
T4	17	6	11	
Nodal status (N)				0.402
N0	12	3	9	
N1	8	4	4	
N2	9	3	6	
N3	21	4	17	

### Increased miR-1228* Expression Suppresses Xenograft Tumor Formation

**Figure 2:**
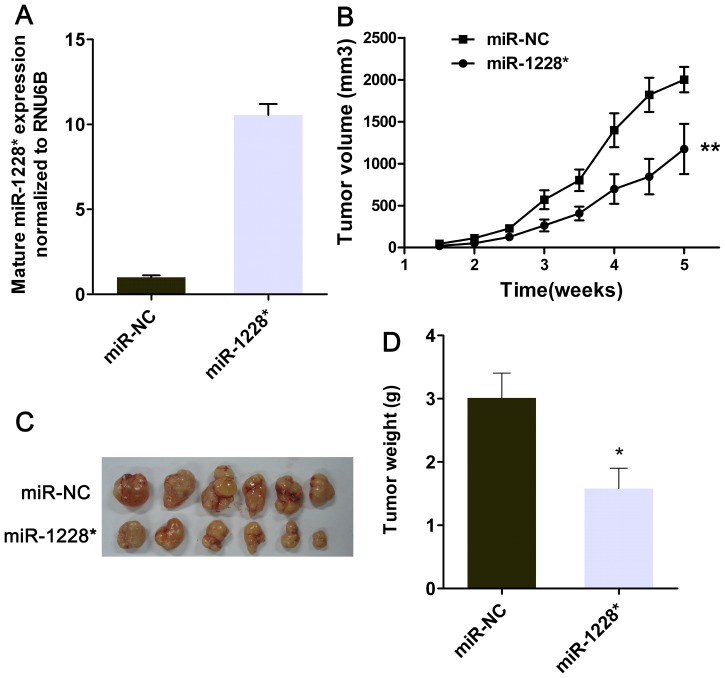
miR-1228* restoration inhibits xenograft tumor formation of gastric cancer cells. (A) miR-1228* was more than ten-fold changes in miR-1228* stable transfected SGC-7901 compared with NC. Results are means ± SEM, n  =  3. (B) Increased miR-1228* expression suppresses xenograft tumor growth. Stable transfection of SGC-7901 cells with miR-1228* or miR-NC were injected subcutaneously into nude mice. The volume of each tumor was measured twice each week. The average volume of tumors developed in nude mice is shown as means ± SEM, n  =  6 per treatment group. The statistical differences between samples were determined by the two-way ANOVA. (C) Compared with the control, the xenografts with miR-1228* overexpression were significantly smaller. The mice were sacrificed 5 weeks after inoculation. Two groups’ photograph of tumors is shown. (D) Tumors from each group were weighed immediately after removal. The tumor weight is indicated as means ± SEM, n  =  6.

In order to assess functional role of miR-1228* in gastric cancer, the optimized miR-1228* stem-loop was cloned into pcDNA6.2-GW/EmGFP vector. Gastric cell line SGC-7901 cells were transfected with pcDNA6.2-GW/EmGFP-miR-1228* or pcDNA6.2-GW/EmGFP-NC vector and stable cell lines were generated and named miR-1228* or miR-NC, respectively. As expected, RT-PCR analysis showed that miR-1228* stable cells had an increase of mature miR-1228* expression when comparing to miR-NC stable cells (Fig. 2A). There was no morphologic change in miR-1228* stable-transfected cells compared with nontransfected cells. SGC-7901 cells with or without transfection of miR-1228* were implanted subcutaneously into the flanks of nude mice to evaluate the potential effects of miR-1228* on gastric cancer growth *in vivo*. Overexpression of miR-1228* significantly suppressed the tumor growth (Fig. 2B). By the end of the experimental period, the size and wet weight of tumors with miR-1228* overexpression was significantly smaller and lower than that of the control group (Fig. 2C–D). Thus, the data indicates that miR-1228* inhibits xenograft tumor growth of gastric cancer cells *in vivo*.

### NF-κB Activation is Responsible for the Lower Expression of miR-1228* in Gastric Cancer

**Figure 3:**
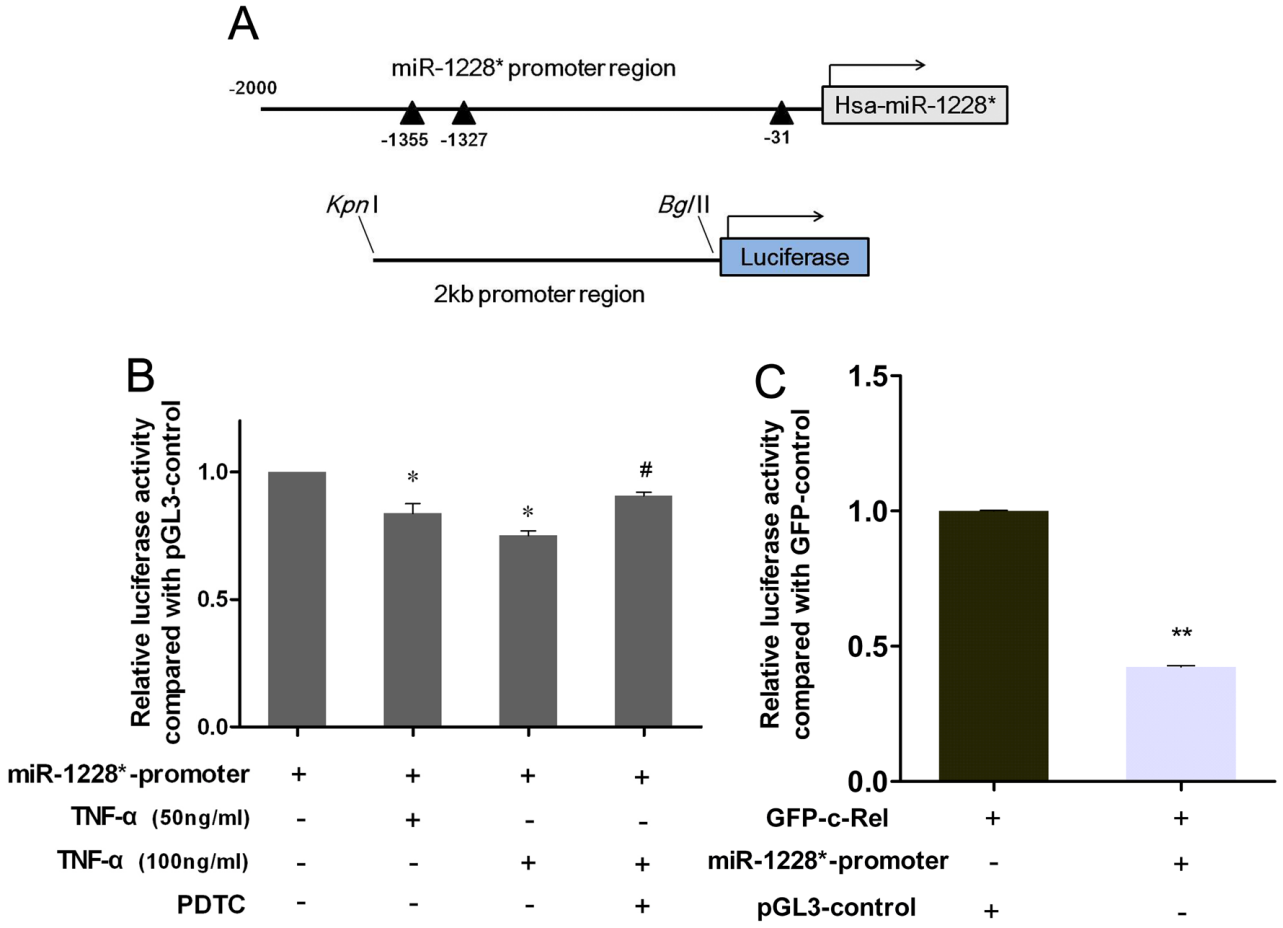
NF-κB activation is responsible for the lower expression of miR-1228*. (A) Schematic representations of the miR-1228* promoter region (the black arrowheads is the c-Rel binding domains), the lower is the pGL3-miR-1228*-promoter construct. (B) 24 hours after transfection with pGL3-miR-1228*-promoter or pGL3-control, SGC-7901 cells were stimulated with or without TNF-α and/or PDTC for 8 hours, then relative luciferase activity was measured. The luciferase activity was normalized to Renilla luciferase activity expressed by pRL-TK and then compared with the relative luciferase level of pGL3-control. * *P* <0.05 as compared with control. ^#^ *P* <0.05 as compared with TNF-α-treated group. (C) SGC-7901 cells were co-transfected with pGL3-miR-1228*-promoter/pGL3-control and GFP-c-Rel/GFP-control, and the relative luciferase expression was detected 48 hours later. The luciferase activity was normalized to Renilla luciferase activity expressed by pRL-TK and then compared with the relative luciferase level of GFP-control. Data were expressed as means ± SEM, n  =  3.

Recent work has demonstrated that most miRNAs have transcription factor (TF) binding sites and many studies have reported that miRNAs could be regulated by TFs [19]. Here we utilized bioinformatic programs ( Promoter 2.0 and P-match) to identify potential regulators of miRNA-1228* [16], [17]. We found three c-Rel binding domains in the 2-kb miR-1228* promoter region (Fig. 3A). Since c-Rel subunit is a member of the NF-κB family and hyperactivation of NF-κB activity has been shown to be associated with gastric cancer [20], [21]. We hypothesized that NF-κB activation is attributed to the downregulation of miR-1228* in gastric cancer.

To prove our hypothesis, we created a luciferase construct with miR-1228* promoter region and evaluated its activity in response to NF-κB activation. An experiment was performed by culturing SGC-7901 cells for 8 hours in the presence of TNF-α (50 or 100 ng/ml, classic NF-κB pathway activator) and/or pyrrolidine dithiocarbamate (PDTC, 100 µM), a widely used inhibitor of the transcription factor NF-κB [22]. SGC-7901 cells were transfected with pGL3-miR-1228*-promoter or pGL3-control, 24 hours later, transfected SGC-7901 cells were exposed for 8 hours to TNF-α with or without PDTC to investigate whether NF-κB activation regulates miR-1228* promoter activity. We observed that TNF-α treatment markedly inhibited miR-1228* promoter activity and inhibition of NF-κB with PDTC prevented TNF-α-mediated downregulation of the activity levels (Fig. 3B), suggesting that NF-κB may negatively regulate miR-1228* expression. To further determine whether NF-κB subunit c-Rel is responsible for deregulating miR-1228*, we transfected SGC-7901 cells miR-1228*-promoter with or without GFP-c-Rel and observed a significant decrease of luciferase activity in GFP-c-Rel group than control (Fig. 3C). Collectively, data suggests that NF-κB subunits c-Rel functionally contributes to the downregulation of miR-1228* in gastric cancer.

### miR-1228* Suppresses NF-κB Activity and CK2A2 Expression in SGC-7901

**Figure 4:**
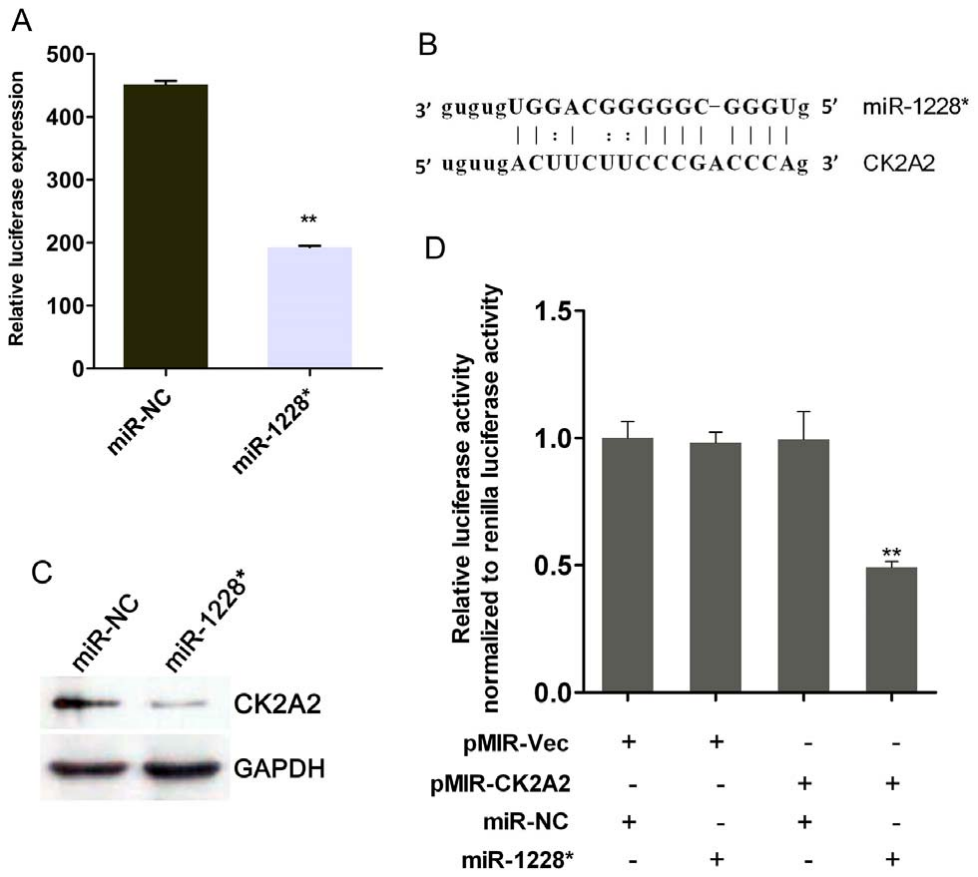
Regulation of NF-κB activity and CK2A2 expression by miR-1228*. (A) SGC-7901-miR-1228* and SGC-7901-miR-NC were transfected with the NF-κB reporter construct, respectively. 48 hours after transfection, the luciferase activity was measured. The luciferase activity was normalized to Renilla luciferase activity. Results are means ± SEM, n  =  3. (B) Schematic graph of the putative binding site of miR-1228* in the CK2A2 predicted by miRanda. (C) Western blot analysis revealed that protein level of CK2A2 in miR-1228* stable overexpression SGC-7901 cells was significantly decreased compared with miR-NC. The level of GAPDH was used as a loading control. (D) miR-1228* significantly reduced the luciferase reading when co-transfected with pMIR-CK2A2 3’UTR plasmid, indicating interaction between miR-1228* and CK2A2 3’UTR at this site. The luciferase activity was normalized to Renilla luciferase activity expressed by pRL-TK. Data were expressed as means ± SEM, n  =  3.

In order to investigate the influence of miR-1228* on the NF-κB signaling pathway, NF-κB reporter constructs were transfected into either miR-1228* or miR-NC stable transfected SGC-7901 cells and the activity of NF-κB luciferase was determined after 48 hours. Our results showed that the activity of NF-κB was significantly decreased by overexpression of miR-1228* (Fig. 4A), indicating a negative feedback loop between miR-1228* expression and NF-κB activity. To further identify downstream targets of miR-1228* in the NF-κB pathway, we performed bioinformatics analysis and found CK2A2 was one of the putative target genes that were predicted. Using miRanda and RNA22 [23], [24], we located one potential binding site for miR-1228* at the 3’UTR of CK2A2 (Fig. 4B). CK2A2 is one subunit of protein kinase CK2 which is involved in the NF-κB pathway [25], [26] and its expression has been observed to be up-regulated in several cancers including gastric cancer [27], [28]. Further investigations on the association between CK2A2 and miR-1228* demonstrated that the protein levels of CK2A2 was concomitantly down-regulated upon miR-1228* stable overexpression in SGC-7901 cells (Fig. 4C). In order to verify whether CK2A2 is a true target of miR-1228*, a segment of the CK2A2 3’UTR containing the binding site was cloned into the 3’UTR of the luciferase gene in pMIR-REPORT plasmid [29]. The fluorescent intensity of the reporter gene was significantly decreased in the group that was co-transfected with pMIR-CK2A2 and miR-1228* compared to the control (Fig. 4D). These results suggested that miR-1228* interacts with the CK2A2 mRNA 3’UTR.

### miR-1228* Inhibits EMT

**Figure 5:**
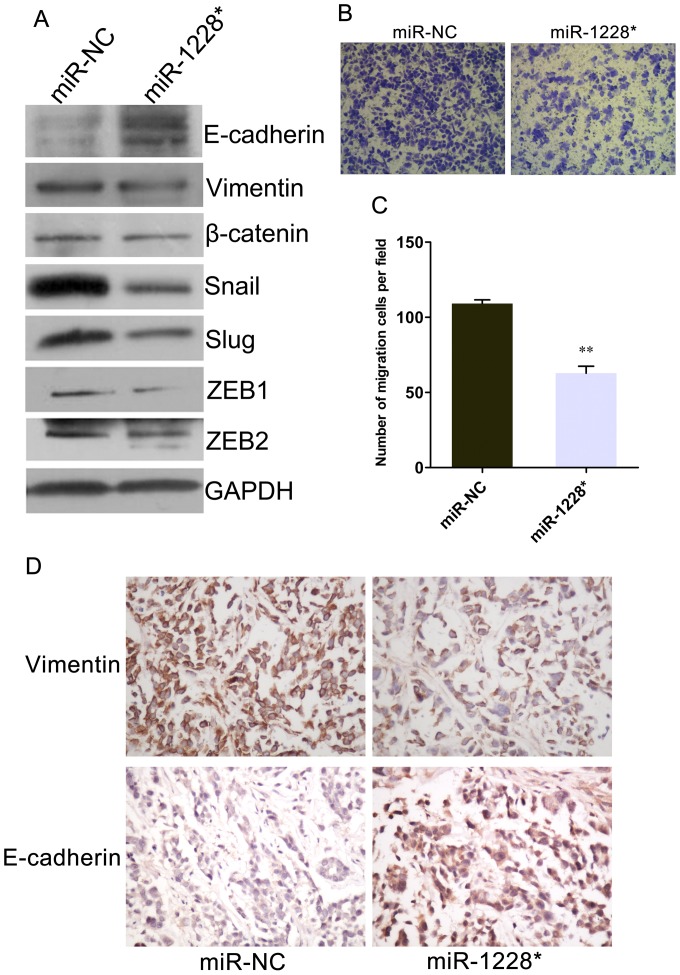
Effect of miR-1228* on EMT in SGC-7901 cells. (A) Western blot analysis of epithelial marker (E-cadherin) and mesenchymal markers (Vimentin, β-catenin, Snail, Slug, and ZEB1/2) in miR-1228* stable transfected SGC-7901 cells and control. (B) Transwell migration assay showed that SGC-7901 cells stable transfected with miR-1228* had lower migratory potential in compare with miR-NC (×100). (C) The relative level of cell migration is presented as the mean ± SEM, based on three independent experiments. (D) Expression of EMT-related markers in xenograft tumor. Immunohistochemical staining indicated decreased Vimentin and increased E-cadherin expression in miR-1228* xenograft tumor compared to the control (×400).

It has been reported that activation of NF-κB play an essential role in EMT for cancer progression [30], [31], and inhibition of NF-κB activity in some tumor cell lines caused a reversal of EMT [32]. Since miR-1228* seems to negatively regulates NF-κB activity, we therefore investigated if miR-1228* could also negatively regulate EMT in gastric cancer. Western blot showed that overexpression of miR-1228* in SGC-7901 cells down-regulated mesenchymal markers (Vimentin, β-catenin, Snail, Slug, and ZEB1/2), while up-regulated epithelial marker E-cadherin (Fig. 5A). Furthermore, miR-1228* overexpression caused a decrease of cell migration (Fig. 5B–C). Immunohistochemical staining was employed to further confirm EMT-related proteins changes of miR-1228* transfected SGC-7901 cells in the xenograft model. It was shown that the expression of Vimentin decreased and the expression of E-cadherin increased when compared with which in control cells (Fig. 5D). The similar findings were observed in another gastric cancer cell line AGS (Fig. S1). The data provide the first evidence that miR-1228* restoration in gastric cancer negatively regulates EMT.

## Discussion

Gastric cancer is the fourth most common malignant tumor worldwide and currently is the second leading cause of cancer-related mortality [33], [34]. Although current practice that combines chemotherapy or radiation therapy with surgical resection has greatly increased the overall survival of gastric cancer patients, the overall 5-year survival rate is still low. Gastric carcinogenesis is considered to be a multifactorial and multistep process that involves the activation of oncogenes and the inactivation of tumor suppressor genes [35].

miRNAs are endogenous non-protein-coding short RNAs that play important roles in cellular physiology, development, and diseases by negatively regulating gene expression. Analyses of miRNA expression profiles have shown that many miRNAs are aberrantly expressed and correlated with tumorigenesis, progression, and prognosis of various haematological and solid tumors including gastric cancer [36]. To identify and elucidate the biological functions of miRNAs deregulation in gastric cancer may help us to better understand the pathogenesis of this deadly disease and provides novel opportunities to develop miRNA-based therapies. For example, miR-221 and miR-222 have been shown to be positively regulating gastric cancer cell proliferation and invasion, suggesting that select inhibition of those miRNAs be beneficial for gastric cancer treatments [37]. miRNAs play a role in the aetiology and pathogenesis of various cancers by targeting a number of oncogenes or tumour suppressors genes. miR-21 is overexpressed in H. pylori-infected gastric mucosa and gastric cancer tissues. Enforced expression of miR-21 increases the invasiveness of gastric cancer cells, RECK is the direct target of miR-21 [38]. miR-218 inhibits the invasion and metastasis of gastric cancer by targeting the Robo1 receptor [39]. Ueda et al [40] identified 22 up-regulated and 13 down-regulated miRNAs in gastric cancer versus non-tumor mucosa, Low expression of let-7g and miR-433 and high expression of miR-214 were associated with unfavorable outcome in overall survival independent of clinical covariates, including depth of invasion, lymph-node metastasis, and stage. miR-375 is frequently down-regulated in gastric cancer and function as a tumor suppressor to regulate gastric cancer cell proliferation potentially by targeting the JAK2 oncogene [10]. Our another study showed miR-1228* was one epithelial cancer-related miRNA (unpublished). However, only few researches of miR-1228* have been reported. Guled et al [41] demonstrated that miR-1228* was highly expressed in malignant mesothelioma samples compared with normal samples. But the biological function of this miRNA has not yet been evaluated. In the present study, we identified that miR-1228* was down-regulated in more than 70% of gastric cancer samples when compared with their nontumor counterparts and provide experimental evidence that it may function as a tumor suppressor through negatively regulating EMT and inhibiting NF-κB activity.

Our data indicated downregulation of miR-1228* in gastric cancer tissues and gastric cancer cell lines. Various molecular mechanisms lead to miRNA dysregulation, such as genetic mutation, epigenetic aberration and deregulated transcriptional activity [42]. TFs could regulate miRNA expression by binding to promoter regions, in either physiological or pathological contexts [43]. To investigate how miR-1228* was deregulated in gastric cancer, we analyzed the 2-kb promoter region upstream of the miR-1228* and found three putative c-Rel binding domains. Although NF-κB is best known for its transcriptional activation function, recent work with p65, RelB and c-Rel demonstrated that NF-κB can down-regulate specific genes [17], [44], [45]. Using a miR-1228* promoter-reporter construct that contained 2-kb fragments of the miR-1228* promoter, we found that NF-κB pathway activator TNF-α decreased miR-1228* promoter activity. And NF-κB inhibitor PDTC remarkably antagonized TNF-α-mediated miR-1228* promoter low activity. Our data also showed that c-Rel was able to binds directly with miR-1228* promoter and negatively regulate its expression. Since c-Rel subunit is a member of the NF-κB family, NF-κB activation phenocopied the effects of c-Rel in regulating miR-1228* expression, suggesting that NF-κB pathway was negatively involved in the downregulation of miR-1228* in gastric cancer. Furthermore, miR-1228* overexpression also resulted in the downregulation of NF-κB activity. Therefore, our data outline a double negative feedback loop between NF-κB and miR-1228*. The exact mechanism of how this negative feedback occurs needs to be further studied.

CK2A2 is one subunit of protein kinase CK2, which is a highly conserved and ubiquitous protein serine/threonine kinase. Overexpression of CK2 has been observed in a number of cancers, including those of the mammary gland, prostate, kidney, lung, head and neck and stomach [27], [46]. Overexpression of CK2 in the mammary glands of transgenic mice causes hyperplasia and dysplasia that eventually lead to adenocarcinomas [47]. CK2 were overexpressed in head and neck squamous cell carcinoma lines and tissues. Knockdown of CK2 subunits differentially inhibited IκBα degradation, NF-κB nuclear localization, phosphorylation, DNA binding, and reporter activity [25]. CK2 plays an important role in Her-2/neu signaling, promoting IκB degradation and NF-κB activation [26]. In this study, we showed that miR-1228* reduced the expression of CK2A2 at the protein level. The luciferase assay with a reporter containing the miR-1228* binding sequence at the 3’UTR of CK2A2 mRNA suggested that miR-1228* directly targets the 3’UTR of CK2A2. Taken together, these data indicate that the suppression of NF-κB activity by miR-1228* may be through targeting CK2A2 expression. EMT is now all known to occur in a variety of diseases including the progression of cancer. It is recognized that EMT is an important process to form diffuse histology and initiate metastasis by enhancing the motility of tumor cells [48]. EMT is regulated by different oncogenic signaling pathways such as AKT and NF-κB [49], [50]. Specifically, activation of NF-κB has been shown to promote EMT while inhibition of NF-κB activity in mesenchymal cell causes a reversal of EMT [30]. Loss of E-cadherin expression and gain of vimentin expression are considered to be the most important molecular markers of EMT [51]. TFs, such as Snail, Slug, ZEB1 and ZEB2, have been shown to have a critical role. miRNAs have key roles in regulating the EMT process. The miR-200 family and miR-205 regulate EMT by targeting ZEB1 and ZEB2, which function as transcriptional repressors of E-cadherin expression, thereby reducing the aggressiveness of cancer cells [52]. Stable expression of miR-155 significantly inhibits cells from undergoing EMT and inhibits the expression of a number of genes related to the induction of EMT [53]. Overexpression of CK2 was involved in the carcinogenesis and development of Colorectal cancer through regulation of EMT-related genes [54]. Activation of CK2 and c-Rel induces EMT via an aryl hydrocarbon receptor and Slug signaling pathway [55]. In our study, restoration of miR-1228* suppressed EMT phenotype and reduced gastric cancer cell migration, this could possibly attribute to miR-1228*-mediated downregulation of NF-κB activity and through targeting CK2A2 expression.

The present work identifies miR-1228* as a negative regulator of NF-κB activity and highlights its role in suppressing EMT and gastric cancer growth, suggests that selective restoration of miR-1228* might be beneficial for gastric cancer therapy.

## Supporting Information

Figure S1 Effect of miR-1228* on EMT in AGS cells. (A) Western blot analysis of epithelial marker E-cadherin and mesenchymal marker Vimentin in miR-1228* stable transfected AGS cells and control. (B) Transwell migration assay showed that AGS cells stable transfected with miR-1228* had lower migratory potential in compare with miR-NC (×100).
(DOC)

## Acknowledgments

The authors thank Dr. Yongliang Zhu in Second Affiliated Hospital of Zhejiang University College of Medicine for technical assistance.
