# Supplementary figures and images for: Restoration of miR-1228* Expression Suppresses Epithelial-Mesenchymal Transition in Gastric Cancer
Source: PLoS One. 2013 Mar 12;8(3):e58637. doi: 10.1371/journal.pone.0058637 (PMC3595239; doi:10.1371/journal.pone.0058637)

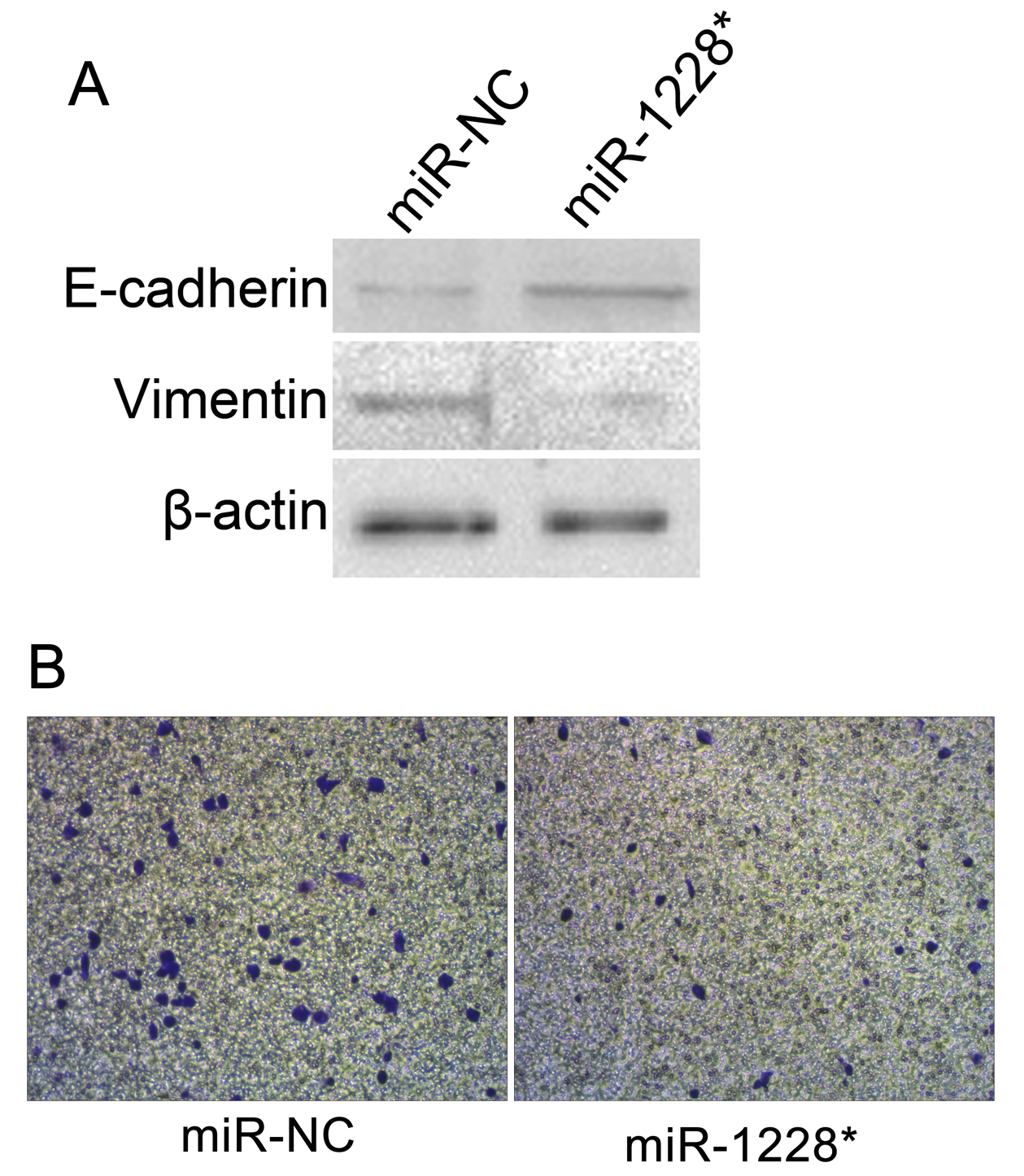

Supplement: Figure S1 — Effect of miR-1228* on EMT in AGS cells. (A) Western blot analysis of epithelial marker E-cadherin and mesenchymal marker Vimentin in miR-1228* stable transfected AGS cells and control. (B) Transwell migration assay showed that AGS cells stable transfected with miR-1228* had lower migratory potential in compare with miR-NC (×100). (DOC) [file pone.0058637.s001.doc]
